# Real‐Time Imaging of Ammonia Release from Single Live Cells via Liquid Crystal Droplets Immobilized on the Cell Membrane

**DOI:** 10.1002/advs.201900778

**Published:** 2019-08-24

**Authors:** Mashooq Khan, Weiwei Li, Sifeng Mao, Syed Niaz Ali Shah, Jin‐Ming Lin

**Affiliations:** ^1^ Department of Chemistry Beijing Key Laboratory of Microanalytical Methods and Instrumentation MOE Key Laboratory of Bioorganic Phosphorus Chemistry & Chemical Biology Tsinghua University Beijing 100084 China

**Keywords:** hyperammonemia, liquid crystals, polymeric microcapsules, single cells, tumor cells

## Abstract

Tumor cells exhibit prominent metabolic alterations through which they acclimatize to their stressful microenvironment. These cells have a high rate of glutaminolysis and release ammonia (NH_3_) as a byproduct, which may function as a diffusible signal among cancer cells and can reveal cellular heterogeneity. E7, a nematic liquid crystal (LC), is doped with 4‐pentyl‐4′‐biphenyl carboxylic acid (PBA) and encapsulated in polymeric microcapsules (P‐E7_PBA_), which are then immobilized on cells in a microfluidic channel. Normal human umbilical vein endothelial cells (HUVECs) and myeloma, human primary glioblastoma (U87), human colon carcinoma (Caco‐2), and human breast adenocarcinoma (MCF‐7) cells are investigated for the release of NH_3_. The P‐E7_PBA_ is able to visualize NH_3_ release from the cell via a radial‐to‐bipolar (R‐B) orientation change, observed through a polarized optical microscope. The various cell lines significantly differ in their response time required for an R‐B change. The mean response times for Caco‐2, U87, and MCF‐7 cells are 277, 155, and 121 s, respectively. NH_3_ release from a single cell captured in a microwell flow chip shows a similar R‐B change. The P‐E7_PBA_ droplets technology could be applied to other multiple targets by functionalizing LCs with different probes.

## Graphical abstract

**Ammonia (NH_3_)** diffuses from the cell to the cellular microenvironment. With a dissociation constant (p*K*a) of 9.3, the NH_3_ + NH_4_^+^ content is 99% composed of NH_4_^+^ ions. The NH_4_^+^ ions interact with the COO^−^‐functionalized liquid‐crystals (LCs), which results in a radial‐to‐bipolar change of the LC droplet. The dashed lines in the droplets show the orientation of the LC molecules.

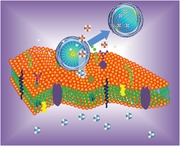

## 1 Introduction

Ammonia (NH_3_) is a toxic cellular byproduct of glutamine metabolism. Enzymes that function in the urea cycle synthesize NH_3_ in vitro. Glutamate dehydrogenase and glutaminase introduce NH_3_ into the urea cycle through the metabolism of l‐glutamate and l‐glutamine, respectively.^1^ The gene mutation, gene expression level, or an inherited deficiency in the activity of these enzymes causes hyperammonemia.^2^ Myeloma is associated with an excess of NH_3_ in the cellular microenvironment. This condition may be due to the reliance of tumor cells on glycolysis for the production of energy, which reduces the requirement for mitochondrial phosphorylation. Due to metabolic reprogramming in cancer cells, mitochondria adopt a new anabolic role to fulfill the high biosynthetic need resulting from cellular proliferation. The mitochondria of tumor cells produce the intermediate α‐ketoglutarate (α‐KG) for the tricarboxylic acid (TCA) cycle.^3^ The α‐KG is generated from glutamine as a result of a two deamination reactions, and NH_3_ is released as a byproduct.^4^ The NH_3_ readily diffuses across the cell membrane, following the chemical potential gradient. Thus, the real‐time imaging of NH_3_ release from cells to the cellular microenvironment could play a vital role in exploring the behavior and heterogeneity among tumor cells. However, analyzing metabolites released from cells to the surroundings is difficult due to rapid fluctuations and dilution to ultralow concentrations,^5^ which obscures the cellular activities. The prominent techniques of spectroscopy and mass spectrometry focus on the bulk medium and can retrieve information from lysed cells; these techniques are aimed at large molecular weight compounds.^6^ Our design of polymer‐encapsulated liquid crystal (LC) droplets immobilized on the cell membrane can provide a fast and an efficient way to selectively track a target molecule or ion released from the cells, or from a single cell, to the surroundings.

The LCs are anisotropic liquid phases and can be best described as a liquid that possesses orientating molecular order.^7^ Nematic phase is the simplest LC phase formed by low molecular weight, rod‐shaped molecules, such as 4‐pentyl‐4′‐cyanobiphenyl (5CB) and E7 (an LC exhibits nematic phase at *T* = 18–60 °C).^8^ LC molecules in a nematic phase align along a single common vector known as the “nematic director.” This directional arrangement results in LC anisotropy, which affects the magnetic susceptibility, birefringence, and dynamic behavior of the nematic phase.^9^ Long‐range ordering minimizes the elastic energy of the system and extends the surface orientation of the mesogen to the bulk LC molecules. Due to low interfacial energy, the physiochemical changes at the interface could induce ordering to a distance of 10^5^ molecular length.^10^ These properties, combined with their anisotropic physical properties, allow the LCs to amplify and transduce molecular events into an optical output, which can be observed through a polarized optical microscope (POM). LCs have widespread applications in liquid crystal display. In the last two decades, LCs have been utilized for the development of actuators and sensors. The orientation of LCs has been coupled to proteins,^11^ lipids,^12^ nucleic acids,^13^ pathogens,^14^ externally added surfactants through LC incorporated on the cells,^15^ and other biomolecules^16^ in the aqueous medium. However, to our knowledge, an LC‐platform has not yet been designed for imaging metabolite release from cells.

**Figure 1:**
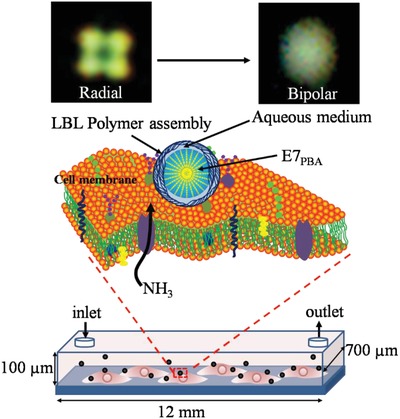
Schematic of immobilized P‐E7_PBA_ droplets on cells cultured in a microfluidic channel. The dimensions of the channel were 12 mm (length), 100 µm (height), and 700 µm (width). NH_3_ released from the cell results in a radial‐to‐bipolar change of the E7_PBA_ encapsulated in the polymeric microcapsule.

In this study, the LC E7 was doped with 4‐pentylbipenyl‐4ʹ‐carboxylic acid (PBA (E7_PBA_)) and was then filled into polymeric microcapsules (P‐E7_PBA_). E7 was used rather than 5CB due to the need to facilitate a high nematic‐to‐isotropic transition. P‐E7_PBA_ droplets were immobilized on cells that were cultured in a microfluidic channel. Live imaging of NH_3_ release from the cells/a single cell was performed through a radial‐to‐bipolar (R‐B) orientation change of P‐E7_PBA_ under cross‐polarization (**Figure** 1). Parallel and perpendicular orientations of LC molecules in a 3D morphology are referred to as bipolar and radial, respectively. When observed through a POM under cross‐polarization, the radial orientation exhibits a single point of defect in the center, while bipolar orientation has two points of defect at the poles. P‐E7_PBA_ has the advantageous features of a controlled size (2–3 µm) to prevent endocytosis, easy immobilization on cell membranes, selective detection of NH_3_ released from cells, high sensitivity, and easy detection through POM. Additionally, the polymer assembly acts as a semipermeable membrane^15^ that allows small molecules to pass through and prevents large molecules from contact with E7; this also avoids contact between E7 and the cell membrane, preventing harm to the cells.

## 2 Results and Discussion

### 2.1 Synthesis and Optical Characterization of P‐E7_PBA_

**Figure 2:**
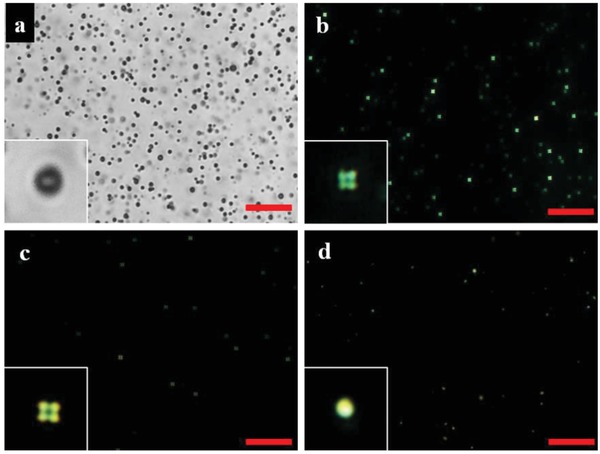
POM images of P‐E7_PBA_ in a) aqueous medium under bright field and in b) aqueous medium and c) MEM, under cross‐polarization. d) P‐E7 in aqueous medium under cross‐polarization. The inset images are not to scale. The scale bars are 10 µm. P‐E7_PBA_: E7 doped with PBA and encapsulated in the polymeric microcapsule. P‐E7: E7 without PBA, encapsulated in the polymeric microcapsule.

Monodispersed polystyrene (PS)‐beads were obtained via dispersion polymerization of styrene, initiator, and stabilizer. The scanning electron microscopy (SEM) and optical microscopy images (Figure S1a, Supporting Information) show that the diameter of the PS beads was ≈2 µm. The PS beads were utilized as a template for the deposition of layer‐by‐layer assembly of polystyrene sulfonate (PSS) and polyallylamine (PAAm) (Figure S1b, Supporting Information). Subsequently, the PS beads were etched to obtain polymeric microcapsules, which were filled with E7_PBA_, to finally obtain P‐E7_PBA_. PBA is an amphiphilic molecule exhibiting a hydrophobic skeleton and hydrophilic carboxylic acid (COOH) functionality. In addition, PBA has a chemical structure resembling 5CB, which is a major component (51%) of E7 (Figure S2, Supporting Information). **Figure** 2a,b shows the microscopy images of P‐E7_PBA_ in an aqueous medium under bright field and cross polarization, respectively. P‐E7_PBA_ exhibited a radial configuration in aqueous medium. A similar radial configuration of P‐E7_PBA_ was observed (Figure 2c) in minimal essential medium (MEM; cell culture medium without supplementation of fetal bovine serum (FBS)). The doped PBA in E7 self‐assembled at the E7/aqueous interface, directing the hydrophilic COOH group toward the aqueous medium and leaving the hydrophobic part embedded in E7. COOH groups deprotonate at physiological pH, increasing the charge density at the E7/aqueous interface, which results in the radial orientation of E7. To clarify the optimum amount of PBA for the preparation of P‐E7_PBA_, E7 was doped with different amounts of PBA. A clear radial configuration was observed with PBA = 10 µg per 100 µL of E7, which was utilized for the subsequent experiments. Furthermore, E7 without PBA exhibited a bipolar configuration (Figure 2d), which confirmed that the radial orientation of P‐E7_PBA_ was due to PBA. To investigate the best droplet texture, P‐E7_PBA_ droplets were observed in the presence of different lambda (λ) plates, in addition to crossed‐polarization (Figure S3, Supporting Information). Comparatively, the radial configuration of the P‐E7_PBA_ under cross‐polarization without a λ‐plate was more visible than in the presence a λ‐plate. Therefore, all of the images of the P‐E7_PBA_ configuration were captured under cross‐polarization only.

### 2.2 Cell Compatibility of P‐E7_PBA_

**Figure 3:**
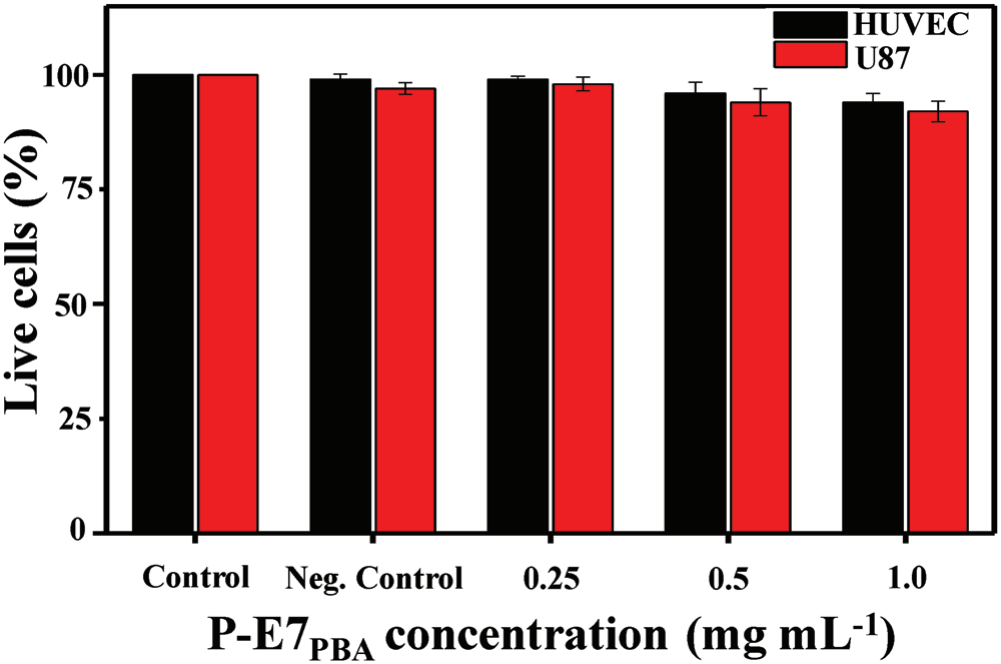
Cell viability in the different P‐E7_PBA_ concentrations. The positive control and negative (Neg.) control were cells without P‐E7_PBA_ treatment and cells with added E7_PBA_, respectively.

The cell compatibility of P‐E7_PBA_ droplets was observed on human umbilical vein endothelial cells (HUVEC) and human primary glioblastoma cells (U87) at different P‐E7_PBA_ concentrations. The cell viability was calculated by Equation (1), where the areas of the green (live) and red (dead) cells were obtained from the fluorescence images (Figure S4a,b, Supporting Information) using Image‐Pro software. **Figure** 3 shows that relative to the control (cells cultured in medium without the addition of P‐E7_PBA_), ≈99% of the cells were viable at a P‐E7_PBA_ concentration of 0.25 mg mL^−1^. The viability decreased to 98% when the concentration was 0.5 mg mL^−1^. At high concentration (1 mg mL^−1^), the P‐E7_PBA_ accumulated on the cell surface in such a way that the cells were not visible, yet 94% of the cells were viable, which suggested the cell compatibility of the P‐E7_PBA_ droplets. Similarly, the cell viability was ≈99% in the negative control (naked E7_PBA_), which may be due to poor dispersion and nonattachment of the E7_PBA_ on the cell membrane. We choose the P‐E7_PBA_ concentration of 0.25 mg mL^−1^ for further experiments because the higher concentrations might have deteriorated the normal cellular activity

(1) $$\text{cell}\;\text{viability}\;\;\left(\%\right)=\frac{{\text{Area}}_{\text{green}}}{{\text{Area}}_{\text{green}+\text{red}}}\;\times \;100$$

### 2.3 Optimization of Extracellular pH

**Figure 4:**
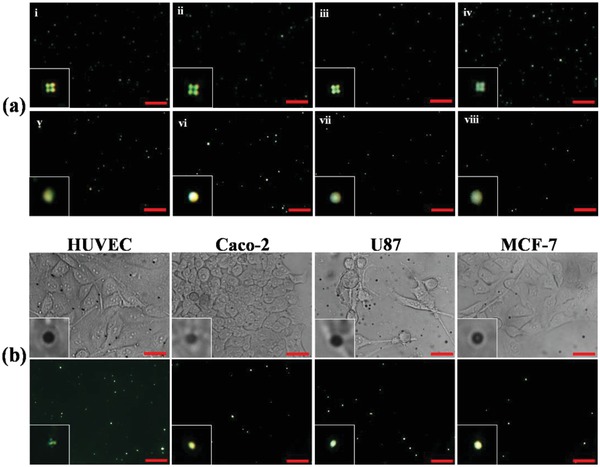
a) POM images of P‐E7_PBA_ at pH i) 8, ii) 7.6, iii) 7.2, iv) 6.8, v) 6.4, and vi) 6 under cross‐polarization; and P‐E7 in MEM at pH vii) 8 and viii) 6. b) Bright field and cross‐polarization microscopy images of P‐E7_PBA_ in the cellular microenvironment of different cell lines. The inset images are not to scale. The scale bars are 10 µm.

The pH responsiveness of the P‐E7_PBA_ droplets was observed in MEM (without supplementation of FBS) at different pH values ranging from 6 to 8 (**Figure** 4ai–vi). The P‐E7_PBA_ droplets exhibited radial configuration at pH ≥ 7. A mixed radial and bipolar configuration was observed at pH = 6.8, while the bipolar configuration became more visible at pH < 6.8. Moreover, the P‐E7_PBA_ droplets showed a reversible R‐B and B‐R configuration by alternative addition of a basic (pH = 7.5) and an acidic (pH = 6.5) solution (Figure S5, Supporting Information). The R‐B change at acidic pH was attributed to the protonation of the PBA COO^−^ to COOH. Doping LCs with molecules containing pH‐sensitive functional groups makes the LC orientation pH‐responsive.^17^ Polyacrylic acid (PAA)‐functionalized 5CB droplets were previously used for the detection of glucose via the glucose oxidase‐catalyzed reaction of glucose, where the proton released from the enzymatic reaction protonated the COO^−^ pendent groups of PAA and caused the R‐B change of 5CB.^18^ P‐E7 was further tested in MEM (pH 8 and 6). The initial bipolar orientation was maintained at both pH values (Figure 4avii–viii), which suggested that the pH responsiveness of P‐E7_PBA_ was due to PBA.

The extracellular pH of normal cells ranges from 7.2 to 7.4, while that of cancerous cells ranges from 6.2 to 6.9.^19^ Therefore, to apply P‐E7_PBA_ droplets for the imaging of NH_3_, the experimental conditions were optimized to equate the extracellular pH. A 0.5 µL aliquot of P‐E7_PBA_ droplets in MEM was introduced onto HUVEC, human colon carcinoma (Caco‐2), U87, or human breast adenocarcinoma (MCF‐7) cells that were cultured in microfluidic channels. Each channel contained ≈800 cells. The radial orientation of the droplets was maintained in the HUVECs, while the R‐B change was observed in the channels containing Caco‐2, U87, and MCF‐7 cells (Figure 4b). The R‐B change was attributed to the acidic pH of the tumor cells. Therefore, the P‐E7_PBA_ droplets were tested in MEM supplemented with lactic acid (1 × 10^−6^ m), ascorbic acid (1 × 10^−6^ m), or a mixture of acidic amino acids (1 × 10^−6^ m). In each case an R‐B change was observed (Figure S6, Supporting Information). P‐E7_PBA_ was then introduced to the same concentration of these analytes in a mixed solution (1:1) of MEM and PBS (pH = 7.4). The radial configuration of P‐E7_PBA_ was maintained, which indicated that PBS prevented the pH change due to these analytes. It was concluded that the P‐E7_PBA_ R‐B change can be prevented via the protonation of acidic species in the cellular microenvironment under controlled experimental pH values. Furthermore, the impact of the mixture solution on the cell activity was evaluated. The U87 cells were separately cultured in MEM and MEM + PBS (1:1) for 3 h in a humid environment, with 95% air and 5% CO_2_ at 37 °C. Relative to the 100% cell viability in MEM, all cells were similarly viable in the mixture solution (Figure S6c, Supporting Information). Therefore, the mixture solution does not appear to disturb normal cellular behavior due to the short analysis time of this technique. Consequently, the imaging of NH_3_ released from the cells was performed in a MEM + PBS (1:1) mixture at pH = 7.4.

### 2.4 Imaging of NH_3_

The efficiency of P‐E7_PBA_ droplets for NH_3_ detection was analyzed in MEM + PBS (1:1) supplemented with aqueous ammonia at different concentrations (0.3× 10^−6^–1.5 × 10^−6^ m) and pH = 7.4. The radial orientation was maintained until an NH_3_ concentration of 0.3 × 10^−6^ m, while an R‐B change became visible at an NH_3_ concentration ≥ 1 × 10^−6^ m (Figure S7a, Supporting Information). The P‐E7_PBA_ was tested in an aqueous NH_4_Cl solution. A similar R‐B orientation change was observed. Furthermore, the P‐E7_DTAB_ (E7 doped with DTAB (dodecyl triamine bromide, a cationic surfactant)) was subjected to aqueous NH_3_ and aqueous NH_4_Cl solutions. The radial orientation of the P‐E7_DTAB_ was maintained (Figure S7b, Supporting Information), suggesting that PBA was responsible for the R‐B change in the presence of NH_4_^+^ ions. The kinetic transfiguration of P‐E7_PBA_ could be an indication of the NH_3_ concentration in a medium. Figure S8a in the Supporting Information shows the R‐B change of P‐E7_PBA_ as a function of time at different NH_3_ concentrations (1 × 10^−6^–2.5 × 10^−6^ m). In the plot, an inferred value of 10% was assigned to the point of initiation of the R‐B change at different concentrations. The speed of the transfiguration was dependent on the amount of NH_3_. At a high NH_3_ concentration, there was a faster R‐B change in the biphenyl carboxylic, whereas at a low concentration, the change occurred slowly. This is quite significant because the P‐E7_PBA_ can respond to the NH_3_ concentration not only in an on–off manner but also in a quantitative relationship that can be derived from the standard curve of NH_3_ concentration against time (Figure S8b, Supporting Information). The standard curve for the point of initiation of the R‐B change could be applied for the NH_3_ concentrations to those when the P‐E7_PBA_ does not achieve a 100% R‐B change.

The selectivity of P‐E7_PBA_ for NH_3_ detection was evaluated in the presence of metal ions (Na^+^, K^+^, Ca^2+^, and Fe^2+^) and biomolecules (glucose, glutamine, and FBS) in MEM + PBS at pH = 7.4. The radial configuration of P‐E7_PBA_ was maintained in 10 × 10^−6^ m solutions of Na^+^, K^+^, glucose, glutamine, and FBS (Figure S9, Supporting Information), while a slight radial‐to‐preradial configuration change was observed in the presence of Ca^2+^ and Fe^2+^. This change was attributed to the linkage of Ca^2+^ or Fe^2+^ with the COO‾ groups of two neighboring PBA molecules. The linkage may tilt the PBA molecules, resulting in a preradial configuration. Next, 0.7 × 10^−6^ or 1 × 10^−6^ m NH_3_ was added to complex mixture solutions containing 2 × 10^−6^ m each of Na^+^, K^+^, Ca^2+^, Fe^2+^, glucose, glutamine, and FBS. Similar to the pure NH_3_ solutions, a slight configuration change in the presence of the solution that contained 0.7 × 10^−6^ m NH_3_ and a complete R‐B change was observed in the solution containing 1 × 10^−6^ m NH_3_. The R‐B change in the presence of NH_3_ may be due to its lipophilic nature. Compared to the other cations, the NH_4_^+^ ion may easily accumulate on the surface of P‐E7_PBA_, and in addition to electrostatic interactions it forms hydrogen bonds between the neighboring PBA molecules.

**Figure 5:**
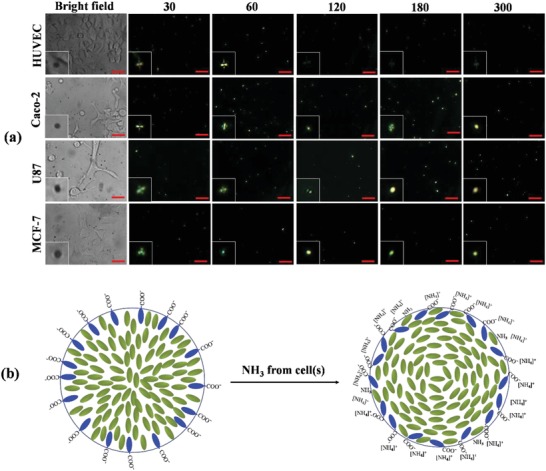
a) POM images of P‐E7_PBA_ droplets immobilized on cells as a function of time. The numerals represent time in s. The inset images are not to scale. The scale bars are 10 µm. b) Schematic representation of E7_PBA_ configuration before and after the interaction of NH_3_ + NH_4_^+^ at the interface. The NH_3_ diffuses from the cell to the cellular microenvironment. With a dissociation constant (p*K*a) of 9.3, the NH_3_ + NH_4_^+^ content is 99% composed of NH_4_^+^ ions. NH_4_^+^ interacts with the COO^−^‐functionalized LCs, reducing the net charge density at the LC/aqueous interface, which results in the R‐B orientation change of the P‐E7_PBA_ droplets.

For live imaging of NH_3_ released from cells, P‐E7_PBA_ droplets (0.25 mg mL^−1^) were immobilized on the cell membrane. Movies S1 and S2 in the Supporting Information show the P‐E7_PBA_ droplets before and after immobilization on the cell membrane, respectively. The number of P‐E7_PBA_ droplets per cell can be increased by increasing the concentration of the droplets. In the present experimental setup it was difficult to control the density of the P‐E7_PBA_ droplets immobilized on a single cell. However, the use of droplet microfluidics or other experimental advancements may provide a solution to anchor a desired amount of droplets onto a single cell. The radial orientation was maintained on HUVECs, while over time a gradual R‐B change was observed in the P‐E7_PBA_ droplets on Caco‐2, U87, and MCF‐7 cells (**Figure** 5a). The analysis time was recorded after passing 15 µL of (MEM + PBS (1:1)) through the channels for 5 min post immobilization to ensure the complete removal of the prereleased metabolites to the medium. A significant difference in response time (*t*_pass_) for the R‐B change was observed for Caco‐2, U87, and MCF‐7 cells, which followed the order of *t*_pass_ = MCF7 < U87 < Caco‐2. During the R‐B change, a mixed radial and preradial‐like configuration appeared for Caco‐2 at *t*_pass_ = 120 s. The R‐B change became more visible at *t*_pass_ = 180 s. At *t*_pass_ = 30 s the preradial configuration was observed in MCF‐7 cells but not in U87. A clear axial configuration was found for U87 at *t*_pass_ = 120 s, while the axial state did not appear for Caco‐2 and MCF‐7 cells. The sequential orientation changes from radial, preradial, axial, to bipolar can be termed as the reverse‐sequence‐orientation change because the sequential change usually appears during the anchoring of the LC droplets as they change from bipolar to radial.^20^ In a reverse sequence, the point of defect of radial orientation migrates from the center of a droplet, leading to a preradial orientation. The point of defect then extends from the side, drawing an equatorial declination line (axial configuration); subsequently, the line splits and shrinks to both poles, forming the bipolar configuration (Figure S10, Supporting Information). The orientation of the P‐E7_PBA_ droplets was controlled by the net charge density; therefore, the lack of visible appearance of each configuration of P‐E7_PBA_ droplets immobilized on the cell membrane may be due to the small size and high sensitivity of the droplets.

With a dissociation constant (p*K*a) of 9.3, NH_4_^+^ constitutes ≈99% of the total ammonia (NH_3_ + NH_4_^+^) concentration at a physiological pH (7.1–7.5). In its ionized form, NH_4_^+^ is relatively impermeable to cell membranes. At pH >6.8, P‐E7_PBA_ exhibits a radial configuration, and rapid protonation of NH_3_ in the cellular microenvironment causes a constant flow of NH_3_ from the cells to the surroundings, which raises the pH of the cellular microenvironment. Under this condition, P‐E7_PBA_ could remain deprotonated, as shown in Figure 5b. An R‐B change occurred when the net charge density at the LC/aqueous interface decreased.^21^ Previously, homeotropic (radial)‐to‐planar (bipolar) orientation changes of LCs decorated with either quaternized poly(4‐vinyl pyridine),^22^ PSS,^23^ PAA,^24^ or poly(dimethylaminoethyl methacrylate) (PDMAEA)^25^ on a transmission electron microscopy (TEM) grid were observed after complexation with oppositely charged proteins. Similarly, an R‐B change was observed when single‐stranded DNA was adsorbed onto LC decorated with a cationic surfactant,^13^, ^14^ and the change was reversed upon the hybridization of DNA at the LC/aqueous interface.^26^ Hence, the NH_4_^+^ in the cellular microenvironment interacts with the opposite charge COO^−^ of PBA. This reduces the net charge density and forms hydrogen bonding, leading to the R‐B orientation change of P‐E7_PBA_.

**Scheme 1:**
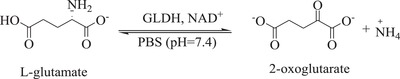
Glutamate dehydrogenase (GLDH)‐catalyzed oxidation of l‐glutamate.

**Figure 6:**
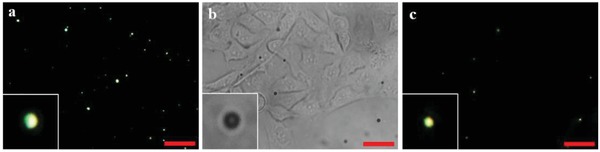
POM images of P‐E7_PBA_ in a) l‐glutamate (10 × 10^−6^ m) in the presence of GDLH (25 µg) and NAD^+^ (2 × 10^−6^ m) in PBS at pH = 7.4; P‐E7_PBA_ immobilized on HUVECs under b) bright field and c) cross‐polarization in the presence of l‐glutamate (10 × 10^−6^ m), GDLH (25 µg), and NAD^+^ (2 × 10^−6^ m) in MEM + PBS at pH = 7.4. The inset images are not to scale. The scale bars are 10 µm. GDLH: glutamate dehydrogenase, NAD^+^: nicotinamide adenine dinucleotide, MEM: minimum essential medium, and PBS: phosphate‐buffered saline.

NH_3_ can be produced via the glutamate dehydrogenase (GLDH)‐catalyzed oxidation of l‐glutamate in the presence of nicotinamide dinucleotide (NAD^+^) cofactor in situ (**Scheme** 1). The P‐E7_PBA_ droplets were tested in the presence of GLDH (10 × 10^−6^ m), l‐glutamate (25 µg), and NAD^+^ (2 × 10^−6^ m) in PBS at pH = 7.4. An R‐B change was observed, which was attributed to the presence of NH_3_ generated from the GLDH‐catalyzed reaction of l‐glutamate (**Figure** 6a). P‐E7_PBA_ immobilized on HUVECs was then subjected to MEM medium containing GLDH, l‐glutamate, and NAD^+^ at pH = 7.4. Similarly, an R‐B transition of the P‐E7_PBA_ droplets was observed (Figure 6b,c). Thus, the NH_3_ released from the cell changed the anchoring condition of the droplets, and in‐depth studies on the droplet configuration could provide a new platform for the quantitative analysis of cellular metabolite.

**Figure 7:**
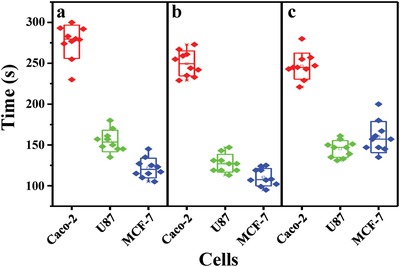
The *t*_pass_ required for R‐B orientation change of P‐E7_PBA_ on different cell lines in MEM + PBS supplemented with l‐glutamine at a) 0 × 10^−6^ m, b) 25 × 10^−6^ m, and c) 50 × 10^−6^ m.

The cellular heterogeneity among the cell lines was explored through the difference in time required (*t*_R‐B_) for an R‐B change of the P‐E7_PBA_ droplets. The *t*_R‐B_ was noted from 10 channels each of Caco‐2, U87, and MCF‐7 cells (**Figure** 7a). An average *t*_R‐B_ of 277, 155, and 121 s was observed for Caco‐2, U87, and MCF‐7 cells, respectively. Estimated from Figure S8b in the Supporting Information (100% R‐B change), the U87, Caco‐2, and MCF‐7 cells released NH_3_ in the range of 0.92 × 10^−6^–1.2 × 10^−6^ m, 1.54 × 10^−6^–2.05 × 10^−6^ m, and 1.91 × 10^−6^–2.64 × 10^−6^ m, respectively. These estimated concentrations may be higher than the actual amount of NH_3_ released from the cells. Furthermore, in comparison to normal cells, the tumor cells exhibited a higher rate of glutamine transport and metabolism.^4^ Normal MEM contains 2 × 10^−6^–4 × 10^−6^ m l‐glutamine. To observe the activity of glutaminase in terms of NH_3_ production, MEM was supplemented with 25 or 50 × 10^−6^ m glutamine (Figure 7b,c). A faster R‐B change was observed for all three cancer cell types at a glutamine concentration of 25 × 10^−6^ m, while retardation was observed for U87 and MCF‐7 at a glutamine concentration of 50 × 10^−6^ m. This retardation may be due to the chemical decomposition of glutamine at high concentrations in the aqueous medium, which produces pyrrolidonecarboxylic acid (PCA) and NH_3_. Therefore, the cyclization of glutamine to PCA at high concentrations results in an increased extracellular NH_3_ concentration. The NH_3_, NH_4_^+^, and H^+^ remained at a dynamic equilibrium due to the rapid protonation and deprotonation of NH_3_.^27^ This led to a reverse diffusion of NH_3_ from the surroundings to the cytoplasm, which was visualized as slowness in the R‐B orientation change. The Berthelot reaction^28^ was used to investigate the NH_3_ release from all cell lines in different conditions (Table S1, Supporting Information), which confirmed that the medium from both U87 and MCF‐7 cells contained less NH_3_ at 50 × 10^−6^ m glutamine compared to normal medium and medium supplemented with 25 × 10^−6^ m glutamine.

### 2.5 Single‐Cell Resolution Imaging of NH_3_

**Figure 8:**
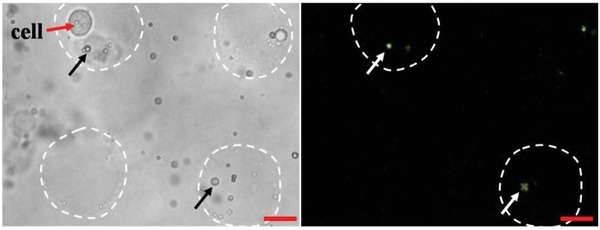
Bright field (left) and cross‐polarization (right) microscopy images of P‐E7_PBA_ droplets immobilized on MCF‐7 cells in a microwell flow chip. The black and white arrows indicate the P‐E7_PBA_ droplets under bright field and cross‐polarization, and the white dotted circles represent the microwells. The scale bars are 10 µm.

The sensitivity of P‐E7_PBA_ at single‐cell resolution was evaluated in a homemade microwell flow chip (Figure S11, Supporting Information). The MCF‐7 cells were cultured in a microwell flow chip. Some of the microwells contained a single cell, while most of the wells contained more than one cell. Optimizing several factors, including cell number (cells mL^−1^), can achieve individual cells per microwell. The NH_3_ release response of P‐E7_PBA_ immobilized on MCF‐7 cells cultured in a microwell flow chip was observed. An R‐B orientation change of the P‐E7_PBA_ droplets was observed in the microwells containing single MCF‐7 cells, while the radial orientation was maintained in neighboring wells that did not contain any cells (**Figure** 8). These results elaborated the utility of P‐E7_PBA_ to detect NH_3_ at single‐cell resolution, and investigating the *t*_R‐B_ for various individual cells could revealed the cellular heterogeneity among identical cell types. Reducing the size of the LC droplets increased the sensitivity to communicate the physicochemical changes at the surface. In this study, we found the ideal size of P‐E7_PBA_ droplets that cannot undergo endocytosis. The miniaturized size of the droplets enabled single‐cell resolution NH_3_ detection, suggesting that the high sensitivity and utility of this method could enable it to be used for evaluating single‐cell heterogeneity.

## 3 Conclusion

This study visualized the NH_3_ release from normal (HUVEC) and myeloma (U87, Caco‐2, and MCF‐7) cells via an R‐B orientation change of the P‐E7_PBA_ droplets under cross‐polarization. P‐E7_PBA_ can easily be anchored on the cell membrane. NH_3_ that diffuses from the cell changes to NH_4_^+^ at physiological pH. The cationic NH_4_^+^ then interacts with the COO^−^ of PBA, which reduces the net charge density at the LC/aqueous interface, resulting in an R‐B orientation change. The *t*_R‐B_ of the R‐B orientation change was the direct indication of NH_3_ concentration release from the cell; this metric was used to differentiate between cell lines and explore cellular heterogeneity. In addition, the P‐E7_PBA_ droplets have the advantages of a controllable size, easy immobilization on the cell membrane, cell compatibility, high sensitivity, and easy detection through POM. By engineering LC‐based probes, this approach can be extended to detect other molecules and ions in the cellular microenvironment and has potential applications in biomedical research.

## 4 Experimental Section

*Synthesis of Microcapsules and Encapsulation of E7_PBA_*: The uniform size PS microbeads were synthesized using a previously reported method^29^ with modifications. Briefly, a mixture of ethanol and deionized water (DIW) (9:1) was heated at 80 °C for 15 min in a three‐neck flask. Poly(4‐vinylpyrrolidone) (*M*_n_ = 40 kDa, 0.4 g) was then added and stirred for 15 min, styrene (5 mL) was then slowly added to the reaction medium over the course of 5 min. Finally, 2,2‐azobisisobutyronitrile (20 mg) was introduced as a radical initiator, and the reaction was refluxed at 80 °C for 24 h with continued stirring. The PS bead suspension was cooled to room temperature and centrifuged at 7000 rpm for 5 min. The PS beads were washed twice with ethanol and dried to powder at room temperature.

PSS and polyallylamine (PAAm) solutions (1 wt%) were prepared in ethanol:DIW (1:1). The PS beads (50 mg) were dispersed in PSS solution (20 mL) in a centrifuge tube and agitated for 12 h at room temperature to allow the adsorption of PSS on the PS bead surface. The suspension was then centrifuged at 5000 rpm for 5 min. To remove the loosely adsorbed PSS, the PS beads were redispersed in ethanol and washed before the deposition of the second layer. After the deposition of the first layer, the PS beads were redispersed in PAAm (20 mL) solution and shaken for 30 min, followed by centrifugation at 5000 rpm for 2 min. Thus, the layer‐by‐layer assembly was achieved by repetitive alternative depositions of PSS and PAAm until the desired number of layers (ten layers each) was deposited onto the PS beads. After every four bilayers, the PS beads were transferred to a new centrifuge tube to prevent aggregation. The coated particles were dispersed in DIW and treated with toluene for 15 min to etch the PS beads.

The prepared polymeric microcapsules were washed twice with ethanol and centrifuged. The resulting pellets were then suspended in 1 mL of ethanol and shaken gently. E7 (100 µL) doped with pentyl‐biphenyl carboxylic acid (PBA, 10 µg) (E7_PBA_) was added to the polymeric pellets, and the resulting mixture was placed on an automated shaker plate at room temperature for 15 h in a closed container. The ethanol and E7_PBA_ form an isotropic phase that can penetrate more readily through the walls of polyelectrolyte‐based microcapsules.^15^ The ethanol was then slowly evaporated by keeping the centrifuge tube uncapped for 24 h. Over this time period, E7_PBA_ was observed to return to the nematic phase, resulting in trapping of the LC within the microcapsules. Excess E7_PBA_ was then removed by centrifugation, and the remaining LC‐filled microcapsules were extracted into deionized water with gentle shaking. The LC‐filled capsules were then characterized using a polarized optical microscope. The polymeric microcapsule‐filled E7_PBA_ (P‐E7_PBA_) was then sterilized under UV‐radiation and suspended in the respective medium for further experiments.

*Cell Culture*: HUVEC and MCF‐7 cells were cultured in RPMI‐1640 supplemented with 10% FBS and 1% penicillin and streptomycin (1:1). Similarly, the U87 and Caco‐2 cells were cultured in MEM supplemented with 10% FBS and 1% penicillin and streptomycin (1:1). All cell types were cultured in Petri dishes for 2–3 days in a humidified atmosphere of 95% air and 5% CO_2_ at 37 °C. When the cells were in the exponential growth phase they were detached from the Petri dishes with 0.25% trypsin and resuspended in corresponding cell culture medium (unless otherwise mentioned) for further experiments.

*Cell Compatibility Test*: HUVEC and U87 cells were separately seeded in 24‐well plates in their corresponding growth medium and were allowed to adhere for 3 h. The culture medium was then withdrawn from the wells using a micropipette. P‐E7_PBA_ (sterilized overnight under UV light) was suspended in cell culture medium at different concentrations (0.25, 0.5, and 1 mg mL^−1^) and then added to the cell‐containing wells (≈1 × 10^6^ cells mL^−1^). Cells without added P‐E7_PBA_ droplets and those with added E7_PBA_ were used as the positive and negative control, respectively. The cells were grown in a humidified environment of 95% air and 5% CO_2_ at 37 °C for 12 h. The medium was then removed and the cells were gently rinsed with PBS. Calcein AM (10 µL) and propidium iodide (PI (15 µL)) were mixed in PBS (5 mL) and added to the cells. The cells were allowed to stain for 25 min in a humidified environment of 95% air and 5% CO_2_ at 37 °C. The PBS was then gently removed with a pipette, the cells were rinsed with fresh PBS, and the cell medium was added to allow a longer time for efficient analysis. The live/dead cells were then observed using a fluorescence microscope.

*Microfluidic Channel Preparation*: The PDMS microfluidic chips with straight channels were prepared using the photolithography technique. A silicon wafer (SW) was washed in gently boiling piranha solution (caution: piranha is corrosive and must be handled with care) for 1 h and subsequently washed with DIW and dried under nitrogen flow. The SU‐8 2050 negative photoresist was uniformly spin coated onto the wafer at a speed of 1200 rpm. The coated SW was baked at 65 °C for 20 min. A photomask was then placed on the SW, and it was exposed to UV light for 3 min. The SW was then baked for 10 min and developed with an SU‐8 developer, which generated a clear mold.

The PDMS prepolymer and the initiator were mixed at a recommended mass ratio of 10:1 and poured into the mold. Bubbles were removed by vacuum, and the polymer was baked at 65 °C for 6 h. The polymerized PDMS was then peeled from the mold, cut, punched and sealed with glass substrate via oxygen plasma. The microfluidic chip used in this experiment has four straight channels. Each channel has a length, width, and height of 12 mm, 700 µm, and 100 µm, respectively. The volume of each channel was ≈0.84 µL.

*Cell Culture in Microfluidic Channels*: The cells were first digested with trypsin, centrifuged, and resuspended in fresh culture medium. A cell density of ≈1 × 10^6^ cells mL^−1^ was achieved by counting with a hemocytometer. The cell suspension was injected into the microchannel from the inlet and incubated for 12 h under a flow of 95% air and 5% CO_2_ at 37 °C. The inlets and outlets of the channels were covered by culture medium to prevent evaporation. The evaporation could change the salt concentration of the medium and affect cell viability. The medium was then refreshed, and the adhered cells in the channels were utilized for further analysis.

*Optimizing the Extracellular pH*: First, the pH‐responsiveness of the P‐E7_PBA_ droplets was tested in MEM at different pH values (8, 7.6, 7.2, 6.8, 6.4, and 6). The pH of the MEM medium was adjusted using citrate buffer. The P‐E7_PBA_ droplets (0.25 mg mL^−1^) were dispersed in the media samples, and the orientation of E7 was observed using POM.

A 0.5 µL aliquot of P‐E7_PBA_ dispersed in culture medium (pH = ≈7.4) was then injected into the channels containing cells. The orientation of the P‐E7_PBA_ droplets in the channels and on the cells was observed under cross‐polarization. All experiments were performed in a sterile environment to ensure the microbial‐free cells.

Next, to negate the effect of common pH‐reducing species, the MEM was supplemented with lactic acid (1 × 10^−6^ m), ascorbic acid (1 × 10^−6^ m), or amino acids (1 × 10^−6^ m). Similarly, the same concentration of lactic acid, ascorbic acid, or amino acids was added to MEM + PBS (1:1). The orientation of the P‐E7_PBA_ droplets was then observed in the MEM and MEM + PBS (1:1) samples containing these analytes. The P‐E7_PBA_ droplets were dispersed in the MEM + PBS (1:1) mixture for further experiments.

*Immobilization of P‐E7_PBA_ Droplets on Cell Membrane*: Prior to immobilization, the P‐E7_PBA_ was sterilized overnight under UV light to avoid microbial contamination. The P‐E7_PBA_ was then dispersed in MEM + PBS (0.25 mg mL^−1^) and injected into a channel containing the cells under analysis. The P‐E7_PBA_ was allowed to immobilize on the cell membrane for 10 min. The amino groups on the outer layer of the LBL assembly facilitated immobilization due to the negative charges on the cell membrane. Subsequently, fresh MEM + PBS was introduced into the channels to wash out the nonimmobilized droplets. During the process, a few droplets were also attached to the glass under the channels, which may have been due to the interaction of slight negative charges on the glass surface with the amino groups on the outer layer of the polymeric microcapsule.

*Imaging of NH_3_*: The P‐E7_PBA_ droplets were tested in aqueous NH_3_ solutions of different concentrations (0.1‐1.5 × 10^−6^ m) in PBS at pH = 7.4. For NH_3_ imaging in the cellular microenvironment, the cells with the immobilized P‐E7_PBA_ in a microfluidic channel were first washed by flowing MEM + PBS (1:1) to achieve the complete replacement of channel medium with fresh medium. This process washed out all the cell debris and ions that were released from the cells to the medium. PBS was used to avoid pH changes in the medium. After immobilization, the fresh mixture was flowed a few times into the channels. The change in P‐E7_PBA_ droplet orientation was then observed with the *t*_pass_. During the ammonia imaging, the pH of the medium was maintained at 7.4.

*Preparation of Microwell Flow Chip*: A mold of the SW was obtained using a photolithography technique, as previously reported.^30^ Briefly, the SU‐8 2050 negative photoresist was spin coated onto a piranha‐washed SW at 1500 rpm and then baked at 65 °C for 20 min. Next, a glass photomask was placed on the coated SW and exposed to UV light for 7 min. Baking was again performed at 65 °C for 10 min, and the coated SW was then developed with an SU‐8 developer to obtain a clear mold.

Subsequently, a 3% aqueous solution of agarose gel was heated at 150 °C until the solution became transparent. The solution was then poured in the mold and was slowly cooled. The gel structure containing microwells was gently peeled from the mold surface. Each microwell had a diameter of 25 µm and a depth of 120 µm. A similar‐sized PDMS cavity was obtained from the mold design with the same procedure. The PDMS layer was placed over an agarose gel to obtain a microwell flow cell for subsequent experiments.

*Cell culture in the Microwell Flow Chip and Imaging of NH_3_ at Single‐Cell Resolution*: The microwell flow cells were washed with PBS several times and sterilized under UV light for 3 h. The cells from the culture medium were then detached using 0.25% trypsin, washed with PBS, centrifuged, and redispersed in fresh medium at a final cell density of ≈1 × 10^6^ cells mL^−1^. The cells were then injected into the inlet of the microwell flow chip, and the drag force along the direction of the flow helped to accumulate the cells into the microwells. The cells in the microwell flow cell were cultured for 3 h in 95% air and 5% CO_2_ at 37 °C.

Sterilized P‐E7_PBA_ was dispersed in a mixture of PBS and cell culture medium (0.25 mg mL^−1^) at pH = 7.4. The cells in the microwell flow device were washed several times with a mixture of PBS and cell culture medium. Gentle washing was performed because slightly harsh treatment could damage or detach the cells from the microwells. The P‐E7_PBA_ suspension was flowed into the microwell flow device. Because of the hydrogel surface, the droplets were also attached onto surfaces other than the microwells. The R‐B configuration change of P‐E7_PBA_ was observed with *t*_pass_. The cross‐polarization state of hydrogels cannot be observed as it can for LC in a glass microfluidic channel. Therefore, for the hydrogel‐based microwell flow chip, it was necessary to adjust the cross‐polarization angle during analysis.

*Analysis of NH_3_ in the Cell Culture Medium*: The presence of NH_3_ in the culture medium as a function of time and temperature was investigated using the Berthelot method. The HUVEC, Caco‐2, U87, and MCF‐7 cells at a density of ≈1 × 10^6^ cells mL^−1^ were cultured at room temperature and 37 °C in Petri dishes. The media samples were collected with a *t*_pass_ of 0.5 and 3 h. The Berthelot reagent was prepared as previously reported.^28^ Briefly, aqueous reagent A consisted of phenol (5 g) and nitroprusside (25 mg) in 500 mL of solution, and aqueous reagent B consisted of NaOH (2.5 g) and sodium hypochlorite (NaOCl, 4.2 mL) in 500 mL of solution.

An equal volume (0.5 mL) of reagent A and reagent B were mixed in a vial. Aqueous NH_3_ solution was then added to the mixture and the color was developed for 30 min. A standard curve at different aqueous NH_3_ concentrations, 0.5, 1, 1.5, and 2 × 10^−6^ m, against the maximum absorbance wavelength (λ_max_) = 633 nm was obtained using UV–vis spectrophotometry. The Berthelot reagent was used as a reference solution.

Similarly, the medium sample was added to the reagent A and B mixture, and the color was developed for 30 min. The concentration of NH_3_ was calculated from the absorption at λ_max_ = 633 nm. Culture medium treated with Berthelot reagent in the corresponding condition was used as a reference solution. Due to the instrument detection limit, the standard addition/dilution method was also applied.

## Conflict of Interest

The authors declare no conflict of interest.

## Supporting information

Supplementary

Supplementary

Supplementary
