# Supplementary material for: Real‐Time Imaging of Ammonia Release from Single Live Cells via Liquid Crystal Droplets Immobilized on the Cell Membrane
Source: Adv Sci (Weinh). 2019 Aug 24;6(20):1900778. doi: 10.1002/advs.201900778 (PMC6794618; doi:10.1002/advs.201900778)
Supplement: Supplementary file 1 — Supplementary [file ADVS-6-1900778-s002.pdf]

Copyright WILEY-VCH Verlag GmbH & Co. KGaA, 69469 Weinheim, Germany, 2019.

ADVANCED SCIENCE
Open Access

Supporting Information

for *Adv. Sci.,* DOI: 10.1002/advs.201900778

Real-Time Imaging of Ammonia Release from Single Live Cells via Liquid Crystal Droplets Immobilized on the Cell Membrane

*Mashooq Khan, Weiwei Li, Sifeng Mao, Syed Niaz Ali Shah, and Jin-Ming Lin**

Copyright WILEY-VCH Verlag GmbH & Co. KGaA, 69469 Weinheim, Germany, 2019.

Supporting Information

Real-time imaging of ammonia release from single live cells via liquid crystal droplets immobilized on the cell membrane

Mashooq Khan[‡], *Weiwei Li*[‡], *Sifeng Mao, Syed Niaz Ali Shah, and Jin-Ming Lin**

Department of Chemistry, Beijing Key Laboratory of Microanalytical Methods and Instrumentation, MOE Key Laboratory of Bioorganic Phosphorus Chemistry & Chemical Biology, Tsinghua University, Beijing, 100084, China.

*Corresponding Author Email: jmlin@mail.tsinghua.edu.cn

[‡]These authors contributed equally.

Materials

E7 (a nematic liquid crystal at room temperature $T_{nematic\,=}$ 18-60 °C, Instec, Shanghai, China), 4-pently-4-biphenyl carboxylic acid (PBA, Wako Pure Chemical Industry, Japan), L-glutamate dehydrogenase, type III, lyophilized powder, ≥ 20 units/mg protein (Sigma), nicotinamide adenine dinucleotide hydrate, from yeast (Sigma-Aldrich), SU-8 2050 Negative photoresist (Newton, MA, USA), PDMS prepolymers and initiators (Dow Corning, Midland, MI, USA), silicon wafers with dimeter=75 mm (Xilika, Tianjin, China), and glass microscope slide (from Solarbio Life Science Corporation, Beijing, China) were used. L-glutamine, L-glutamate, and poly-4-vinylpyrrolidone (Mn=40 kDa) were purchased from Amresco. Ascorbic acid, Toluene, Ethanol, and ammonium chloride were obtained from Beijing Tong Guang Fine Chemicals Company. Phosphate buffer saline (PBS), minimal essential medium (MEM), RPMI 1640 medium, trypsin, penicillin and streptomycin were obtained from Gibco Corporation (NY, USA). Fetal bovine serum was purchased from Tianhang Life Science Corporation (Zhejiang, China). Human umbilical vein endothelial cells (HUVEC), human primary glioblastoma cells (U87), large intestinal cells (Caco-2), and breast cancer cells (MFC-7) were purchased from Cancer Institute & Hospital of the Chinese Academy of Medical Science, Beijing, China. Lactic acid

styrene, 2,2-azobisioburyronitrile (AIBN), polystyrenesulfonate sodium (PSS·Na), ammonia solution, and polyallylamine hydrochloride (PAA·HCl) were obtained from J&K Chemicals, China. Calcein AM and Propidium iodide were purchased from Dojindo Molecular Technologies, Inc. All chemicals were of analytical grades and used without any further purification.

Instrumentations

The scanning electron microscopy images were obtained using SEM (FEI Quanta 200, FEI, Netherland). The polarized optical microscopy images were captured using a CCD camera (Moticam2306, Motic, China) connected to a polarized optical microscope (BA310Pol+EPI, Motic, China). The CCD camera provides a resolution of 2048×1536 at frame rate of 13-14 FPS; and the different configurations of P-E7$_{PBA}$ droplets were easily distinguishable at any angle and orientation of the droplets under crossed-polarizers. Spin-coater (KW-4A, Microelectronics Center, Chinese Academy of Sciences), oxygen plasma (PDC-32G, Harrick Plasma, USA). UV-visible spectrophotometer (UV-3900s, Hitachi, Japan), and Fluorescent microscope (Leica DMI 4000B, Wetzler, Germany) were used during experiments.

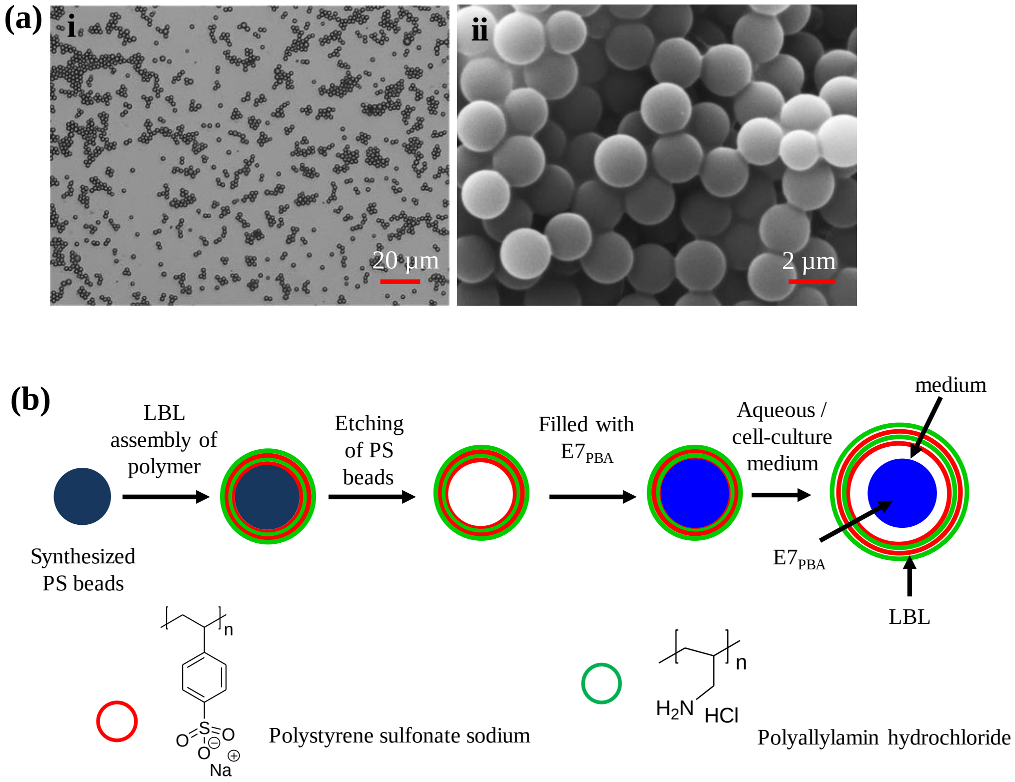

Figure S1. (a) (i) Optical, and (ii) SEM images of PS beads. (b) encapsulation of E7$_{PBA}$ in layer-by-layer assembly of PSS•Na and PAA•HCl. PS: polystyrene, E7$_{PBA}$ (E7 liquid-crystal doped

with 4-pentyl-4´-biphenylcarboxylic acid, PSS•Na: polystyrene sulfonate sodium, PAA•HCl: polyallylamine hydrocholoride.

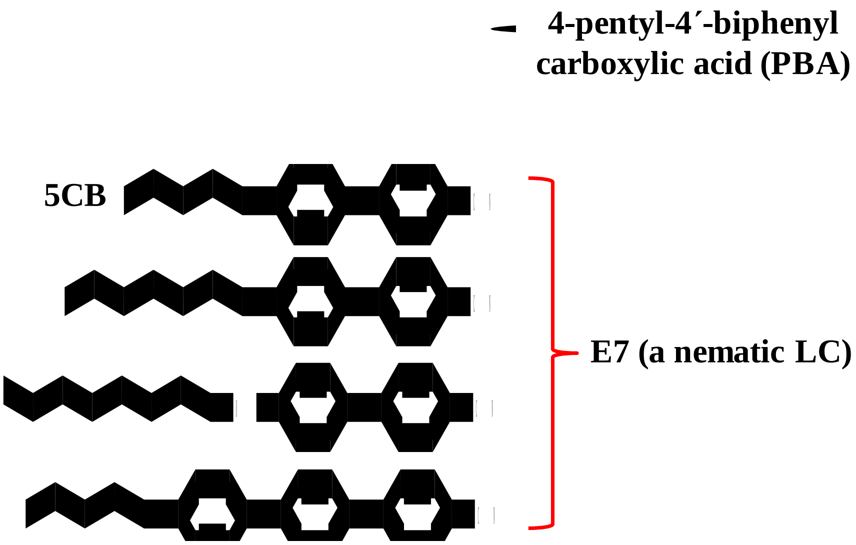

Figure S2. Chemical structure of PBA and E7.

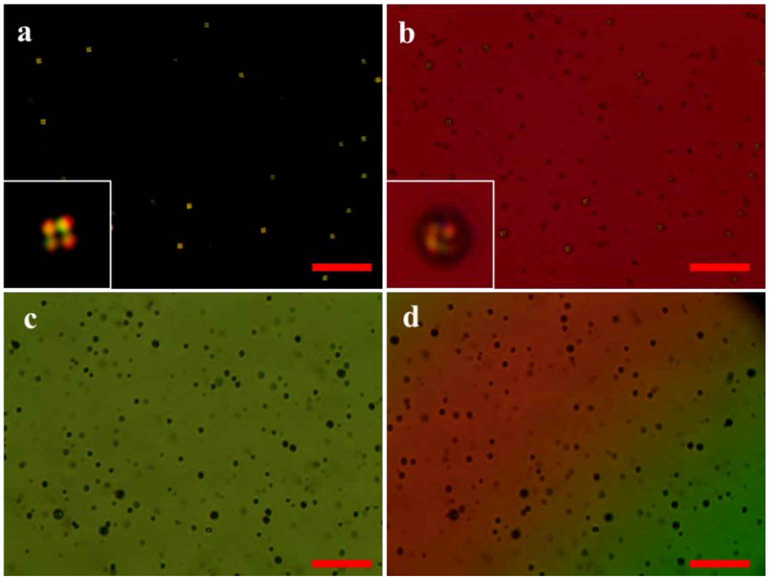

Figure S3. POM images of P-E7$_{PBA}$ observed under cross polarizers (a) without a lambda (λ) plate, and with a (b) λ-plate, (c) ¼ λ-plate, and (d) 1-IV order λ-plate. The scale bars are 10 µm.

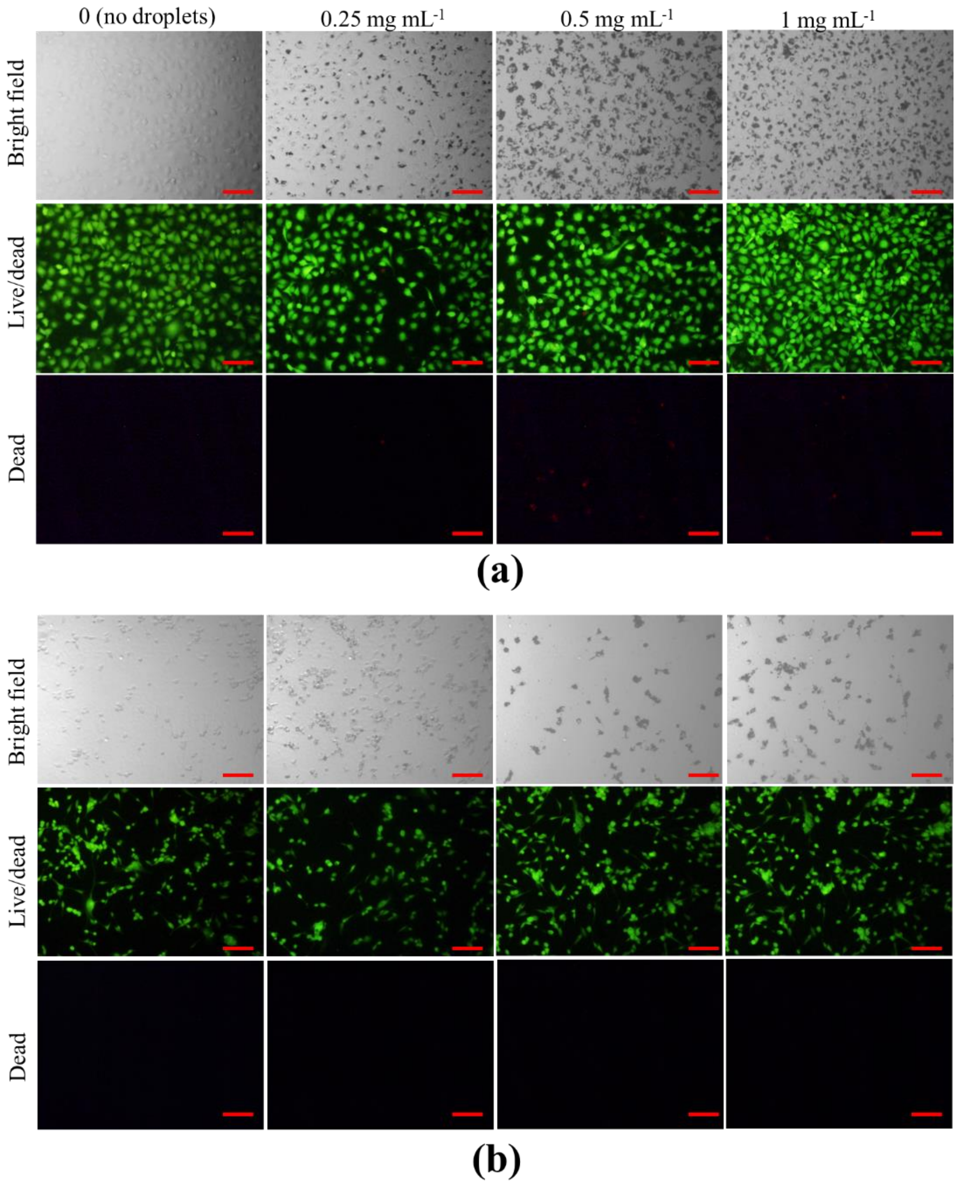

Figure S4. The bright field and fluorescence images of (a) HUVEC, and (b) U87 cells in P-E7$_{PBA}$. The scale bars are 50 μm. HUVEC: human umbilical vein endothelial cells, U87: human primary glioblastoma cells.

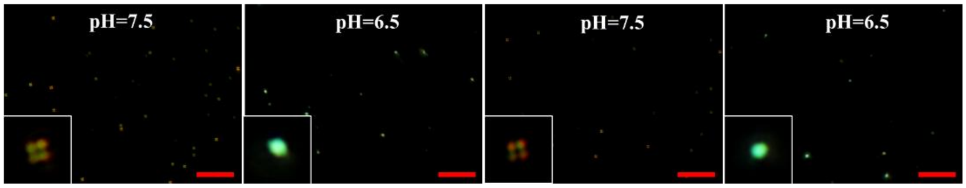

Figure S5. POM images of P-E7$_{PBA}$ by alternative exposure to different pH solutions.

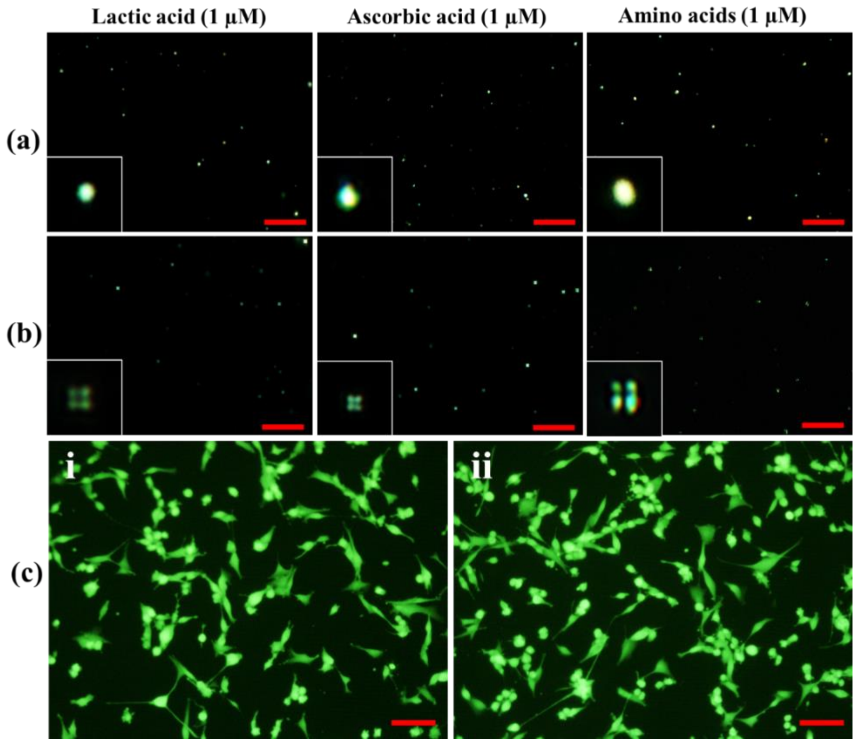

Figure S6. POM images of P-E7$_{PBA}$ in (a) MEM and (b) MEM+PBS (1:1), at pH 7.4. Inset images are not to scale. The scale bars are 10 µm. (c) Fluorescent images of U87 cell cultured in (i) MEM, and (ii) MEM+PBS (1:1) under humid environment (air=95 %, CO2=5 %) at 37 °C. The scale bars are 100 µm. MEM: minimum essential medium, PBS: phosphate buffer saline.

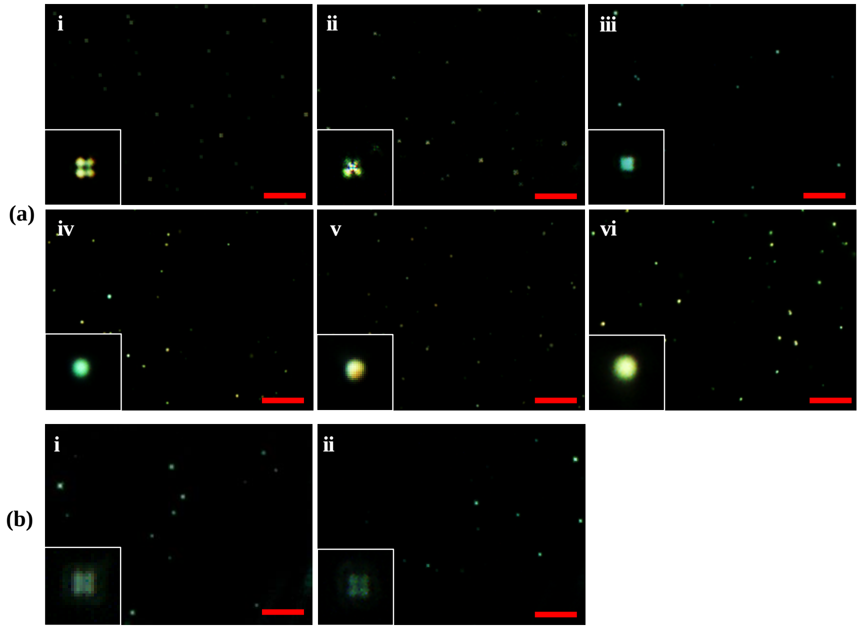

Figure S7. (a) POM images of P-E7$_{PBA}$ in ammonia solution of (i) 0.3, (ii) 0.5, (iii) 0.7, (iv) 1, and (v) 1.5 µM; and (vi) P-E7$_{PBA}$ in NH_4Cl (1 µM) solution and (b) P-E7$_{DTAB}$ in (i) aqueous NH_3 (1 µM) and (ii) NH_4Cl (1 µM) solutions under cross polarizers. The pH of the tested solutions was 7.4. The inset images are not to the scale. The scale bars are 10 µm.

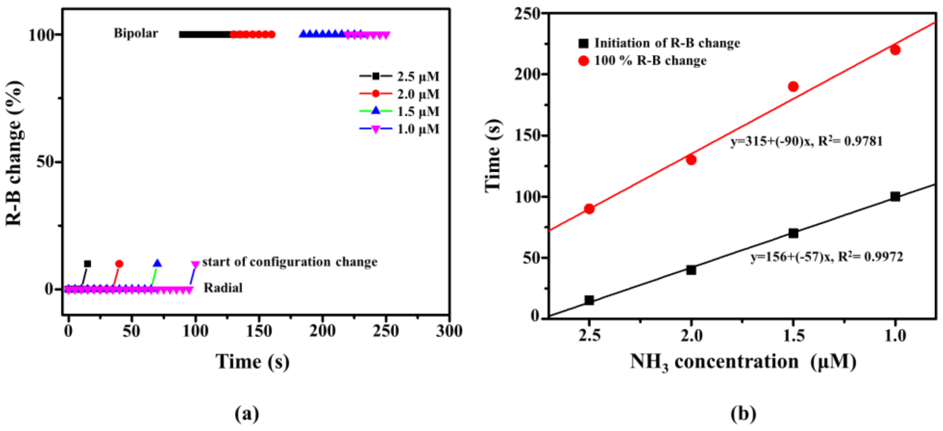

Figure S8. (a) R-B change of P-E7$_{PBA}$ as a function of time at different NH_3 concentration, and (b) plot of t_{R-B} against NH_3 concentration. The standard curve for the point of initiation of R-B change (black line) could be applied for the NH_3 concentrations, at those the P-E7$_{PBA}$ not achieve a 100 % R-B change.

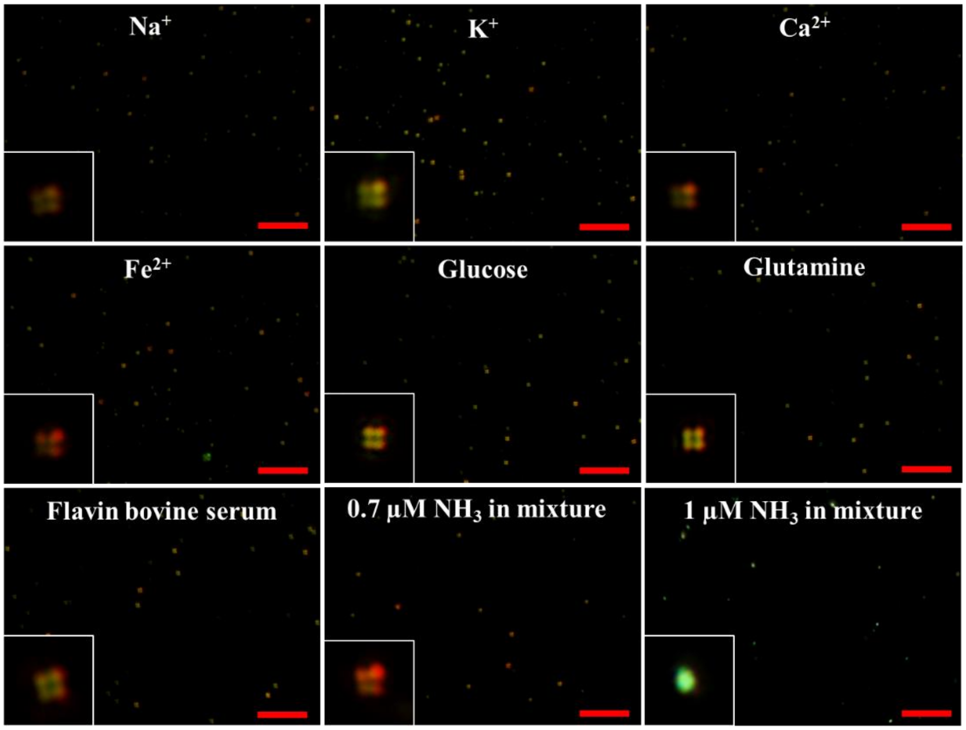

Figure S9. POM images of P-E7$_{PBA}$ in 10 μM solutions of various analytes in MEM+PBS (1:1) at pH=7.4. Inset images are not to scale. The scale bars are 10 μm.

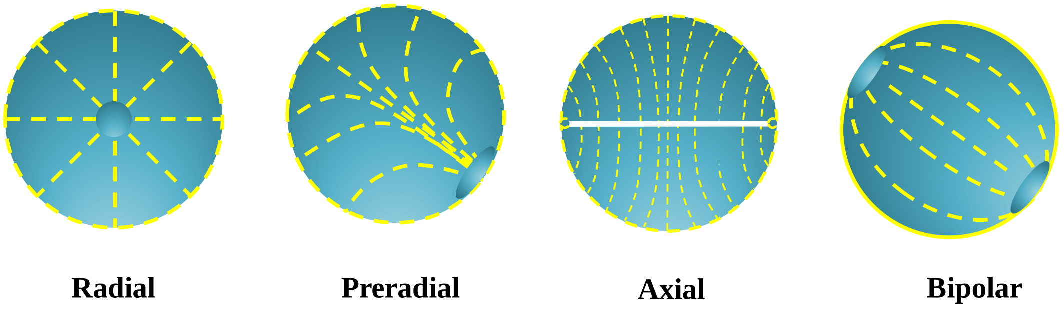

Figure S10. Orientation of LC droplet under different anchoring conditions.

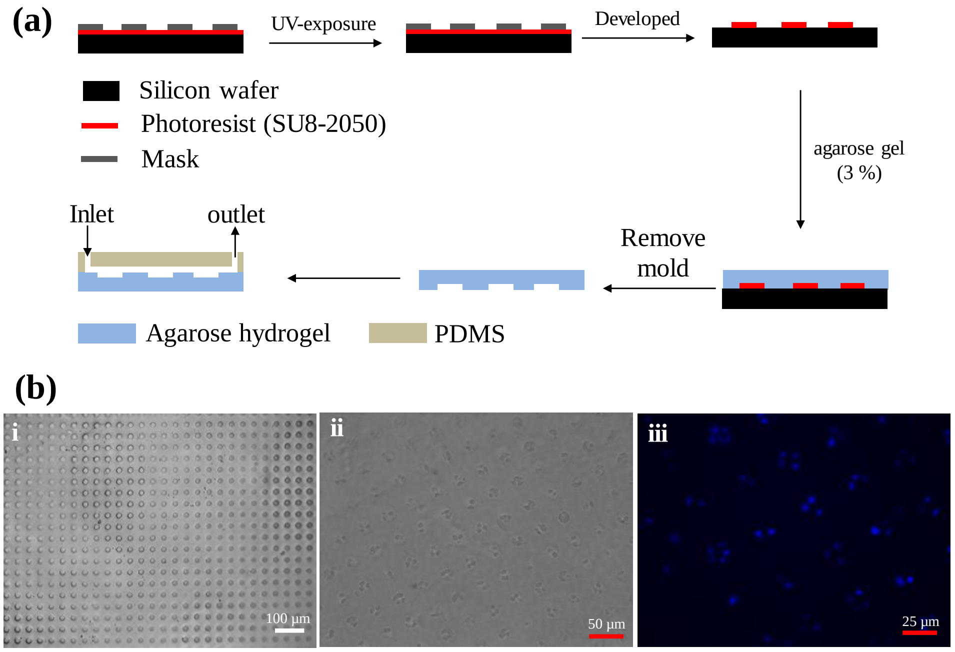

Figure S11. (a) Fabrication of micro-wells flow device. (b) bright field microscopy images of (i) micro-wells and (ii) cells culture on micro-wells flow device. (iii) fluorescence image cell-culture on micro-wells flow device.

Table S1. Ammonia analysis in cell culture medium using Berthelot's method.

Cells	Time (h)	Ammonia concentration (μM)					
		Room temperature			37 ºC		
		Cell culture medium	Cell culture medium + glutamine (25 μM)	Cell culture medium + glutamine (50 μM)	Cell culture medium	Cell culture medium + glutamine (25 μM)	Cell culture medium + glutamine (50 μM)
HUVEC	0.5	0.5	0.7	1.1	1.1	1.9	2.1
	3	2.4	2.8	3.1	3.2	3.7	3.8
Caco-2	0.5	1.2	1.8	1.9	1.8	2.1	2.2
	3	3.5	4.1	4.3	5.1	5.4	5.7
U87	0.5	1.9	2.9	2.7	3.1	4.3	4.1
	3	7.1	11.6	10.4	9.8	11.5	11.3
MCF-7	0.5	2.7	3.5	3.4	3.2	4.1	4.0
	3	8.5	12.3	11.8	10.5	14.6	14.5

Movies

Movie S1. P-E7$_{PBA}$ droplets injected to the microchannel containing cells before immobilization.

Movie S2. P-E7$_{PBA}$ droplets immobilized on cells.
